# Analysis of the Genome and Mobilome of a Dissimilatory Arsenate Reducing *Aeromonas* sp. O23A Reveals Multiple Mechanisms for Heavy Metal Resistance and Metabolism

**DOI:** 10.3389/fmicb.2017.00936

**Published:** 2017-05-29

**Authors:** Witold Uhrynowski, Przemyslaw Decewicz, Lukasz Dziewit, Monika Radlinska, Pawel S. Krawczyk, Leszek Lipinski, Dorota Adamska, Lukasz Drewniak

**Affiliations:** ^1^Laboratory of Environmental Pollution Analysis, Faculty of Biology, University of WarsawWarsaw, Poland; ^2^Department of Bacterial Genetics, Institute of Microbiology, Faculty of Biology, University of WarsawWarsaw, Poland; ^3^Department of Virology, Institute of Microbiology, Faculty of Biology, University of WarsawWarsaw, Poland; ^4^Institute of Biochemistry and Biophysics, Polish Academy of SciencesWarsaw, Poland

**Keywords:** *Aeromonas* spp., dissimilatory arsenate reduction, heavy metals resistance, genome, mobilome, plasmid

## Abstract

*Aeromonas* spp. are among the most ubiquitous microorganisms, as they have been isolated from different environmental niches including waters, soil, as well as wounds and digestive tracts of poikilothermic animals and humans. Although much attention has been paid to the pathogenicity of Aeromonads, the role of these bacteria in environmentally important processes, such as transformation of heavy metals, remains to be discovered. Therefore, the aim of this study was a detailed genomic characterization of *Aeromonas* sp. O23A, the first representative of this genus capable of dissimilatory arsenate reduction. The strain was isolated from microbial mats from the Zloty Stok mine (SW Poland), an environment strongly contaminated with arsenic. Previous physiological studies indicated that O23A may be involved in both mobilization and immobilization of this metalloid in the environment. To discover the molecular basis of the mechanisms behind the observed abilities, the genome of O23A (∼5.0 Mbp) was sequenced and annotated, and genes for arsenic respiration, heavy metal resistance (*hmr*) and other phenotypic traits, including siderophore production, were identified. The functionality of the indicated gene modules was assessed in a series of minimal inhibitory concentration analyses for various metals and metalloids, as well as mineral dissolution experiments. Interestingly, comparative analyses revealed that O23A is related to a fish pathogen *Aeromonas salmonicida* subsp. *salmonicida* A449 which, however, does not carry genes for arsenic respiration. This indicates that the dissimilatory arsenate reduction ability may have been lost during genome reduction in pathogenic strains, or acquired through horizontal gene transfer. Therefore, particular emphasis was placed upon the mobilome of O23A, consisting of four plasmids, a phage, and numerous transposable elements, which may play a role in the dissemination of *hmr* and arsenic metabolism genes in the environment. The obtained results indicate that *Aeromonas* sp. O23A is well-adapted to the extreme environmental conditions occurring in the Zloty Stok mine. The analysis of genome encoded traits allowed for a better understanding of the mechanisms of adaptation of the strain, also with respect to its presumable role in colonization and remediation of arsenic-contaminated waters, which may never have been discovered based on physiological analyses alone.

## Introduction

Aeromonads are ubiquitous microorganisms found in different environmental niches such as fresh and marine waters, sediments and soil (e.g., Dumontet et al., 2000). Moreover, some *Aeromonas* spp. strains, recognized as opportunistic pathogens, have been isolated from wounds and digestive tracts of poikilothermic animals and mammals, including humans (e.g., Chen et al., 2016; Teunis and Figueras, 2016). Due to health threats related to the infections with these bacteria, many studies focus on determining the antibiotic resistance phenotypes and virulence factors of various *Aeromonas* strains (Rasmussen-Ivey et al., 2016). Much less attention is paid to thorough physiological and genomic analysis of *Aeromonas* spp. that have not been described as pathogenic, but which may be involved in processes beneficial from the environmental point of view, e.g., bioremediation and detoxification (Ogugbue and Sawidis, 2011).

Environmental isolates of *Aeromonas* spp. are known to affect the cycle of inorganic and organic matter in the environment (e.g., Wang et al., 2009; Watts and Lloyd, 2013). This is also the case for arsenic, a metalloid, which constitutes a particular threat to living organisms due to its high toxicity, but to which many microorganisms, including Aeromonads, evolved resistance (Drewniak et al., 2010). Much less common among *Aeromonas* spp. is the ability to directly utilize arsenic species to produce energy, for which dissimilatory arsenate reducing bacteria (DARB) are particularly known (Drewniak et al., 2015). This group of bacteria comprises a number of species belonging to different genera, i.a., *Alkaliphilus*, *Bacillus*, and *Shewanella*. Surprisingly, given the ubiquitousness and versatility of metabolism of *Aeromonas* spp., only two dissimilatory arsenate reducing strains belonging to this genus have so far been described: O23A (Drewniak et al., 2010) and OM4 (Drewniak et al., 2014). Both the strains originate from an ancient gold mine in Zloty Stok (SW Poland).

*Aeromonas* sp. O23A, the first known dissimilatory arsenate reducing Aeromonad, was isolated from bottom sediments and microbial mats found in arsenic-contaminated (∼20,000 μg As/l) effluents from the mine. Preliminary analyses carried out by Drewniak et al. (2010) showed that the strain is capable of arsenate respiration using lactate as the donor of electrons. Further, thorough investigation of the O23A physiology was carried out by Uhrynowski et al. (2017). The study revealed that O23A has a versatile metabolism, as it is capable of both aerobic and anaerobic growth using various electron donors and acceptors. Moreover, the strain shows high tolerance to extreme environmental conditions, such as contamination with heavy metals, both low and high pH (4–11) and temperature (4–42°C). Furthermore, under anaerobic conditions, O23A may be involved in direct release of arsenic from rocks containing As-minerals. Interestingly, physiological studies indicated that O23A may facilitate not only mobilization, but also immobilization of arsenic in the environment. This trait is considered unique among DARB, as they typically promote the release of this metalloid from minerals, and thus increase the level of pollution (Okibe et al., 2014; Drewniak et al., 2015). Moreover, metagenomic analysis showed that O23A is one of the dominant DARB strains within the microbial mats community in the Zloty Stok gold mine (Drewniak et al., 2012). The mats may act as effective filters retaining heavy metal ions and other pollutants flowing through the mine waters (Drewniak et al., 2016), leading to their self-purification.

The results of the physiological studies of *Aeromonas* sp. O23A confirm the key role of the strain in colonization and remediation of arsenic-contaminated waters. However, the genetic basis for all the processes in which O23A is involved is still unknown. Genomic studies may provide information on the already known capabilities of the strain, as well as those which have not yet been discovered based on the physiological analyses. For example, comparison with the fairly well characterized clinical isolates of *Aeromonas* spp. may be crucial in the assessment of risks associated with the potential presence of pathogenicity determinants related to, e.g., biofilm formation (Du et al., 2016) and siderophore production (Tomás, 2012). This may prove important in the application for the *Generally Recognized As Safe* (GRAS) status, lack of which may hinder the actual use of the strain in bioremediation, given its taxonomic relatedness to opportunistic pathogens. Comparative genomic analyses can also provide information about the direction of evolution of *Aeromonas* spp. genomes.

Therefore, the aim of this study was a detailed genomic characterization of *Aeromonas* sp. O23A, supported by physiological analyses, which allowed for: (i) an insight into the adaptation strategies of the strain to extreme environmental conditions, (ii) better understanding of its function within the microbial community and (iii) determination of its potential gene transfer mechanisms and relatedness of various Aeromonads. Genomic analyses were focused on chromosomal and mobilome-encoded heavy metal resistance and metabolism genes, while the physiological analysis was aimed to explore the range of heavy metal resistance and mobilization capabilities. This work, therefore, provides a novel insight into the abilities of *Aeromonas* sp. O23A, identifying and underlining the genetic basis of the biogeochemical changes observed in the environment in which the strain may potentially be involved.

## Materials and Methods

### Media and Growth Conditions

The investigated *Aeromonas* sp. O23A strain was isolated from waters, rock biofilms and bottom sediments from the Zloty Stok gold mine (SW Poland) by Drewniak et al. (2010). The strain was routinely grown under aerobic conditions in LB (Sambrook and Russell, 2001) or minimal salt (MS) medium (Drewniak et al., 2015) supplemented with 5 mM lactate and yeast extract (0.04% w/v) as a source of vitamins, at the temperature of 26 ± 2°C. Optimal growth conditions were determined previously by Uhrynowski et al. (2017).

### DNA Manipulations

#### DNA Isolation

Standard DNA manipulation methods were performed as described by Sambrook and Russell (2001). Total DNA was extracted using a kit (Genomic Mini, A&A Biotechnology) from bacterial cells harvested by centrifugation of an overnight culture carried out in LB medium. Purified DNA was used as the template for genome sequencing. Plasmid DNA was isolated analogously using a kit (Plasmid Mini, A&A Biotechnology) or by CsCl gradient method (Sambrook and Russell, 2001).

#### Genome Sequencing, Assembly and Analysis

Genome assembly was performed using a combination of Illumina short data reads and PacBio RS long reads. Two pair-end libraries with an average insert size of 160 and 460 bp were prepared using the Illumina TruSeq v2 kit and sequenced on an Illumina HiScan with 2 × 100 nt read length. Illumina sequencing yielded 0.561 and 0.89 million reads, respectively, which were first trimmed to remove adaptor sequences using cutadapt (v1.8) and then quality trimmed using sickle2 (mean quality 30, min. length 50 nt). PacBio sequencing data was obtained from the High-Throughput Sequencing Facility at the University of North Carolina (Chapel Hill, United States). Library sequencing with an average insert size of 20 kb resulted in 467,925 reads with the mean length of 1.48 kb (longest read: 23.14 kb). Assembly of the obtained reads was performed using SPAdes (v.3.0.0) – *careful option* with PacBio reads supplied as filtered subreads fasta. This yielded 240 scaffold sequences, with a mean length of 20 kb (longest scaffold – 841.97 kb). The scaffold assembly was verified using sequence reads obtained after PCR amplification of selected genome regions, as well as from the fosmid genome library of O23A created in pNGS FOS (Lucigen), which were sequenced in the DNA Sequencing and Oligonucleotide Synthesis Laboratory (oligo.pl) at the Institute of Biochemistry and Biophysics, Polish Academy of Sciences.

The obtained O23A chromosome sequence was annotated using Prokka 4 (v 1.10) and RAST (Aziz et al., 2008) and deposited in the GenBank database. Automatic annotation results for selected open reading frames (ORFs) were verified manually using Artemis software (Carver et al., 2008) and BLAST programs (Altschul et al., 1997) provided on the National Center for Biotechnology Information (NCBI) website^1^. Metabolic pathways were assigned using Pathologic5 from the Pathway Tools 18.5 (Karp et al., 2011) ran on the Prokka output.

#### Plasmid Annotation and Analysis

Plasmid nucleotide sequences were analyzed using Clone Manager (Sci-Ed8) and Artemis software. Similarity searches were performed using the BLAST programs. The manually annotated sequences of O23A plasmids p1–p4 were deposited in the GenBank database.

### Phage Induction and Analysis

Prophage sequences within the draft genome were identified using PHAge Search Tool (PHAST) (Zhou et al., 2011)^2^. To induce potential prophages in *Aeromonas* sp. O23A, bacterial cells were treated with mitomycin C, a classical inducer of lambdoid prophages. The resulting lysate was purified by PEG precipitation and CsCl density gradient separation. The visible band was collected and analyzed for the presence of phage particles by transmission electron microscopy (TEM). Genomic DNA of the phage was extracted from CsCl-purified viral particles and was subjected to high throughput resequencing. The obtained phage sequence was automatically annotated using the RAST server and the resulting annotations were then thoroughly manually curated. BLASTP and Psi-BLAST algorithms were used for the similarity searches in the NCBI^3^, UniProt^4^ and REBASE (Roberts et al., 2015) databases.

### Heavy Metal Resistance Analyses

Heavy metal resistance capabilities of the strain were analyzed by determining the minimal inhibitory concentrations (MICs) of metal ions that completely inhibited the strain’s growth. Cultures were carried out aerobically in titration plates, in LB medium amended with appropriately diluted stock solutions of analytical grade heavy metal salts (3CdSO_4_ × 8H_2_O; CoSO_4_ × 7H_2_O; HgCl_2_; K_2_Cr_2_O_7_). The growth of the strain was assessed by measuring the changes in the optical density at 600 nm (OD_600__nm_) of the cultures compared to the non-inoculated controls using an automated plate reader (Sunrise, TECAN). Spectrophotometric measurements were carried out at 24-h intervals for 3 days, and changes in OD_600__nm_ > 0.2 throughout the experiment were considered positive results. All experiments were repeated three times. O23A was considered resistant to a given metal if it was capable of growth in the presence of at least: (i) 10 mM of As(V), (ii) 1 mM of As(III), Cd(II), Co(II), Cr(VI), Cu(II), Ni(II) or Zn(II), or (iii) 0.1 mM of Hg(II) (Dziewit et al., 2013). MICs for As(III), As(V), Cu(II), Ni(II), and Zn(II) were determined in the previous study (Uhrynowski et al., 2017).

### Siderophore Production and Mineral Dissolution Analyses

*Aeromonas* sp. O23A cultures for the studies of secondary metabolite production were carried out aerobically on GASN medium (Bultreys and Gheysen, 2000) for 72 h at 26°C as described by (Uhrynowski et al., 2017). Uninoculated medium was used as the control. The cultures were centrifuged and the obtained supernatants were passed through sterile 0.2 μm filters, and used for mineral dissolution experiments. The experiments were carried out in 50 ml tubes containing 40 ml of culture supernatants with the addition of 10 g/l of arsenopyrite or pyrite (approximately 1–2 mm fraction). The minerals were washed with HCl and distilled water to remove the oxidized surface and loose particles, dried, weighed, and sterilized. Samples were collected at the beginning of the experiments, and after 7 days of incubation with vigorous shaking. Iron concentrations were quantitatively analyzed by flame atomic absorption spectrometry (FAAS, AA Solaar M6 Spectrometer, TJA Solutions, Cambridge, United Kingdom), using a set of standard solutions (Merck, Darmstadt, Germany) prepared in 0.5 M HNO_3_. All experimental variants were carried out in duplicate.

### Phylogenetic Analysis

The phylogenetic relationship between 30 validly assigned representatives of the genus *Aeromonas*, whose genomes have been sequenced, was analyzed based on the *rpoB* gene nucleotide sequence similarity. The sequences were aligned using ClustalW software^5^. The obtained alignment was adjusted manually and then used to render a phylogenetic tree. The rooted tree was constructed using the distance matrix calculated by the Neighbor-Joining method (Saitou and Nei, 1987). The evolutionary distances were computed using the Kimura 2-parameter formula (Kimura, 1980). The bootstrap consensus tree was inferred from 1000 replicates (Felsenstein, 1985). All the above evolutionary analyses were conducted using MEGA7 software (Kumar et al., 2016).

### Nucleotide Sequence Accession Numbers

The nucleotide sequences of *Aeromonas* sp. O23A chromosome and extrachromosomal replicons pO23A_p1-p4 have been annotated and deposited in the GenBank database (BioSample: SAMN06812489). The .gbk files may be found in the Supplementary Materials (Supplementary File S1).

## Results and Discussion

### General Features of the *Aeromonas* sp. O23A Genome

**FIGURE 1:**
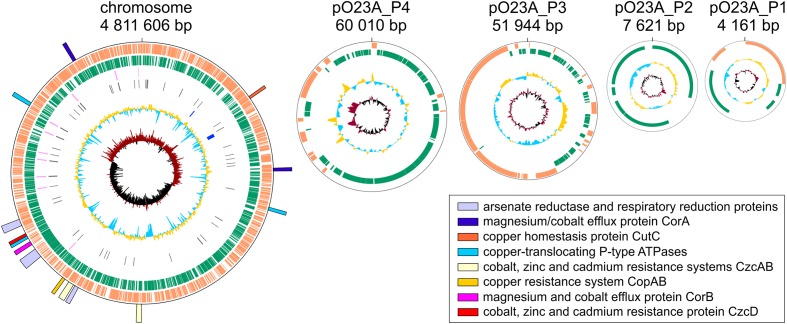
**Physical maps of the chromosome and plasmids of *Aeromonas* sp. O23A**. Open reading frames are represented as orange and green blocks. The innermost circle represents GC skew, whereas the lager circles – GC content, CRISPR regions and RNA genes, respectively. The identified heavy metal resistance and metabolism modules are indicated as colored rectangles, forming the outermost part of the physical map of the O23A chromosome.

The genome of *Aeromonas* sp. O23A is composed of a single circular chromosome of 4,811,606 bp and four circular plasmids: pO23A_P1 (4161 bp), pO23A_P2 (7621 bp), pO23A_P3 (51,944 bp), and pO23A_P4 (60,010 bp). The total size of the genome is 4,935,342 bp. The overall GC content of the chromosome is 58.4%, which is consistent with the other sequenced *Aeromonas* genomes (whose GC content ranges between 58.17% (*A. salmonicida* subsp. *salmonicida* A449) and 62% (*A. hydrophila* 4AK4) (Adamczuk and Dziewit, 2016). The GC contents of the O23A plasmids ranges between 44.7% (pO23A_P3) and 54.3% (pO23A_P1) (**Figure 1** and **Table 1**). Interestingly, the GC contents of all the plasmids are significantly lower than the corresponding value for the chromosome. This phenomenon was also previously noticed in several meta-analyses of plasmids (e.g., Dziewit and Bartosik, 2014), and may be a consequence of the fact that bacteria cannot stably maintain horizontally acquired DNAs, whose GC content is higher than that of the chromosome (Nishida, 2013).

**Table 1:** General features of the *Aeromonas* sp. O23A genome.

General features	Chromosome	pO23A_P1	pO23A_P2	pO23A_P3	pO23A_P4
Size (bp)	4,811,606	4161	7621	51,944	60,010
GC content (%)	58.4	54.3	52.6	44.7	50.5
Coding density (%)	87.5	60.8	79.9	82.5	82.6
Number of genes	4535	7	10	69	76
Number of tRNA genes	118	0	0	0	0
Number of 16S-23S-5S rRNA gene clusters	9 (plus one additional gene for 5S rRNA)	0	0	0	0
Phage regions	1	0	0	0	0

Automatic annotation using the RAST server identified 4710 genes (4535 within the chromosome and 162 within the plasmids) with an average length of 905 bp. The smallest distinguished gene (93 bp), encoding a hypothetical protein, was identified within the plasmid pO23A_P3, and the largest one (14,070 bp) was found within the chromosome and encoded a putative acetyltransferase. Additionally, 118 tRNA genes and 9 clusters of 16S-23S-5S rRNA (plus one additional 5S rRNA-encoding gene) were identified within the *Aeromonas* sp. O23A chromosome (**Table 1**).

A total of 3933 (83.5%) predicted *Aeromonas* sp. O23A proteins were functionally categorized, which allowed for the calculation of the proportions in each COG category (Supplementary Table S1). Over 32% of the proteins with assigned COG numbers were described as involved in the overall cellular metabolism (i.e., categories C, G, E, F, H, and I). Interestingly, as many as 218 of the predicted proteins were classified into the COG category P, which means that they are most probably involved in inorganic ion transport and metabolism (Supplementary Table S1). Moreover, the EC numbers were assigned to over 1000 of the predicted *Aeromonas* sp. O23A proteins. Most of them were classified as transferases (367), then oxidoreductases and hydrolases (212 each), lyases (109), ligases (77), and isomerases (73).

### Phylogeny of *Aeromonas* sp. O23A

**FIGURE 2:**
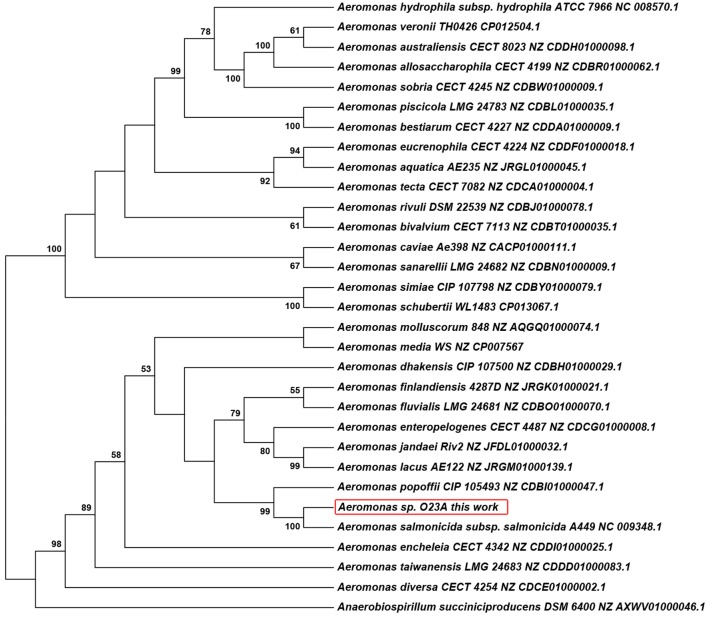
**Phylogenetic tree of *Aeromonas* spp. based on the *rpoB* genes sequences of 30 validly assigned representatives of this genus**. The strain O23A analyzed in this work was indicated with a red frame. The *rpoB* gene sequence of *Anaerobiospirillum succiniciproducens* was used as the root. Accession numbers of the gene sequences used for the phylogenetic analysis are shown. The percentage of replicate trees, in which the associated taxa clustered together in the bootstrap test (1000 replicates) are shown next to the branches. Only values >50% are shown.

Phylogenetic analysis of *rpoB* genes, revealed that *Aeromonas* sp. O23A is most closely related to *Aeromonas salmonicida* subsp. *salmonicida* A449 (**Figure 2**). This is in accordance with the previous phylogenetic analysis based on the 16S rRNA gene sequence comparison of O23A with other Aeromonads (Uhrynowski et al., 2017). The DNA sequences of this gene for these two strains are in 99% identical. Moreover, in the bootstrap test, the strains were clustered together as the closest relatives in all the repeats, which corresponds to very high similarity of the sequences and indicates close phylogenetic relationship between the O23A and A449 strains.

This result may provide information about the origin of O23A. Potentially, the common ancestor of the two strains was brought to the mine waters by salmonid-type fish, which are typical hosts of *A. salmonicida*. The common evolutionary pathway may, however, indicate that O23A is potentially capable of infecting poikilothermic animals, making it unsuitable for use in open bioremediation systems before the genes for potentially dangerous traits are effectively silenced or deleted, (e.g., Darmon and Leach, 2014).

Alternatively, the common ancestor of O23A and A449 might not have been a pathogenic strain, and it became one as a result of gene transfer events between bacteria. This is probable, as the concentration of arsenic in these waters is high and may be toxic to aquatic organisms, preventing their access to the mine. In such a case, O23A may be incapable of infecting fish hosts. The above hypotheses however, have to be confirmed by *in vivo* studies.

### Mobilome Description

To determine the role of horizontal gene transfer in shaping of the *Aeromonas* sp. O23A genome a thorough investigation of the distinguished mobile elements was performed. The mobilome of O23A is quite diversified, as the strain was found to carry four plasmids, one prophage and several putative transposable elements. All of these may play a crucial role in the dissemination of genes in the environment, and are discussed in detail below.

#### Plasmids of O23A

The plasmids of *Aeromonas* sp. O23A range in size from ∼4 kb to over 60 kb. The smallest plasmid (pO23A_p1) comprises seven ORFs, and its backbone consists mainly of a putative RepB protein. The plasmid also encodes two potential transcriptional regulators and six hypothetical proteins of an unknown function.

Plasmid pO23A_p2 (∼7.5 kb) comprises 10 ORFs, including a potential DNA methylase and type II restriction enzyme, with CAGCTG specificity (PvuII). Based on amino-acid sequence comparisons the putative protein is almost identical with the restriction-modification system of *A. hydrophila* SNUFPC-A8. Interestingly, pO23A_p2 carries genes for a membrane protein of the type VI secretion system (T6SS), which may be a potential virulence determinant. Moreover, like all the plasmids of *Aeromonas* sp. O23A, except for pO23A_p1, pO23A_p2 contains a set of genes encoding proteins responsible for mobilization for conjugal transfer (MOB module).

The second-largest plasmid, pO23A_p3 (∼52 kb), apart from the replication protein, contains a partitioning system (ParA, ORF62) and a potential conjugal transfer module. In the case of pO23A_p3, the TRA module may be impaired or otherwise inactive, as the MOB system was also found. The potential function of many predicted proteins encoded by this plasmid remains unknown.

The backbone of the largest plasmid, pO23A_p4 (∼60 kb), consists of two potential replication proteins RepA and RepB, as well as a complete set of transconjugal transfer genes (TRA module). Apart from the TRA module, this plasmid also carries a potential toxin-antitoxin module HicA/HicB (ORF18 and ORF17, respectively). Among the phenotypic modules encoded by the plasmid putative tellurium resistance proteins KlaA protein (ORF44) and KlaB/TelA (ORF45) were found. Therefore, the presence of this replicon in the cells may be beneficial in environments contaminated with this (mildly toxic) metalloid. This plasmid also contains several potential DNA repair proteins, including a UV protection protein (ORF26). ORFs of all the plasmids along with their potential function are listed in Supplementary Tables S2–S5.

The indicated phenotypic modules found within the O23A plasmids, and especially pO23A_p4, may provide the strain with particularly advantageous traits. As the three largest plasmids carry either MOB or TRA modules, they may actively participate in the dissemination of genes via horizontal gene transfer.

#### Phage ΦO23A

Another element of the *Aeromonas* sp. O23A mobilome is the prophage integrated within its chromosome, named ΦO23A. Following induction with mitomycin C, phage particles were analyzed by TEM. TEM images showed that the virion has an icosahedral head approximately 65 nm in diameter and a tail approximately 100 nm long and 40 nm wide (Supplementary Figure S1). These morphological features indicate that the virus belongs to the *Myoviridae* family.

Sequence analysis showed that the genome of the phage ΦO23A was 36 847 bp DNA with a G+C content of 56.7%, which is slightly lower than the average for the host genome (58.4%). The comparison of the restriction profile of the ΦO23A phage DNA (data not shown) with its nucleotide sequence yielded circular restriction maps of the phage genome, suggesting that the DNA molecules of ΦO23A are linear and are circularly permuted. In the *Aeromonas* sp. O23A chromosome the 5′ end of the ΦO23A prophage is preceded by a 112-bp direct repeat of an identical region present in the ΦO23A genome (coordinates 36736 to 36847). This could be the attachment site to the host genome (attB). A part of this region corresponds to the 3′ end of tRNA-Leu gene, which in turn is the last of the three tRNA genes’ cluster [tRNA-Gly(GCC), tRNA-Cys(GCA), tRNA-Leu(TAA)]. The phage ΦO23A presumably utilizes chromosomal sequences encoding a tRNA gene for attachment, like many other temperate bacteriophages (e.g., HP1, ΦLM21). In this case, integration of the phage ΦO23A genome reconstitutes an intact copy of a leucine tRNA(TAA) gene because the 57-nucleotide terminal sequence (coordinates 36791 to 36847) of the ΦO23A right arm is identical with the 3′ end of tRNA-Leu gene. The ΦO23A genome containes 49 putative genes, which share a significant similarity at the protein level with the other sequences in GenBank. Putative functional assignments and significant similarities to the predicted genes are listed in Supplementary Table S6.

Comparison of the nucleotide sequence of ΦO23A with the databank BlastN revealed a high degree of similarity (query coverage 65%, *e*-value 0.0; coordinates 14262–22731) with the P2-like ΦO18P phage of *A. media* (DQ674738, Beilstein and Dreiseikelmann, 2008). Moreover, BLASTP analyses showed that the ΦO23A proteome shares 36 homologs (73.5% of ΦO23A-encoded proteins) with ΦO18P. The shared homologs are encoded in a collinear cluster of genes and include an integrase, transcription regulatory proteins, replication protein A, both terminases, portal and other structural proteins, holin and endolysin. Unique ΦO23A proteins, not having their homologous counterparts in the ΦO18P genome are localized on the left and right ends of the ΦO23A genome. These are: on the left arm ORF1 (putative transcriptional regulator), ORF3 (putative lysogenic conversion protein), ORF4 (CI repressor), ORF6 (Cox-like transcriptional regulator) and on the right arm: ORF46-47 (hypothetical proteins), ORF48 (HNH endonuclease), ORF49 (DNA-cytosine-methyltransferase). It is possible that ORF48 and ORF49 form together a restriction-modification system as similar pairs of homologous genes are found in other genomes (e.g., in *Enterobacter hormaechei* subsp. *steigerwaltii* strain 34998- AKZ84360 and AKZ84359).

The predicted protein product of the ORF49 gene showed similarity to C5-methylcytosine methytransferases (m5C MTases), including M.Eco4255I of *Escherichia coli* O69:H11 07-3763 and M.Eco3763II of *E. coli* O118:H16 07-4255, whose recognition sequence and target motif (GC**C**GGC, the methylated base is underlined) was identified in the genome by SMRT sequencing technology. The specificity of ORF49 was tested by comparative digestion of the pET-O23A_ΦORF49 plasmid DNA, isolated from IPTG-induced and uninduced *E. coli* cultures, with PdiI (GCCGGC), BsuRI (GGCC), Bsh1236I (CGCG), and Hin6I (GCGC) restriction enzymes. The DNA of pET-O23A_ΦORF49 isolated from the induced culture was cleaved by all the tested restriction enzymes, with the exception of PdiI. In contrast, the pET-O23A_ΦORF49, DNA isolated from the non-induced culture was susceptible to all restriction enzymes, including PdiI. The DNAs of ΦO23A, with 31 GCCGGC was completely resistant to PdiI digestion (data not shown). Furthermore, chromosomal DNA of *Aeromonas* sp. O23A was also completely resistant to PdiI digestion indicting that ORF49 is expressed during ΦO23A lysogeny. It is possible that ORF48-encoded putative HNH nuclease is also active during the temperate state of the ΦO23A phage and together they constitute protection against infection of their host by other phages or foreign DNA.

#### Other Mobile Elements

Apart from 4 plasmids and a prophage, the mobilome of *Aeromonas* sp. O23A comprises a number of putative transposable elements. In total 22 genes encoding transposases of transposable elements were identified. According to the ISfinder database (Siguier et al., 2006), they belong to 8 families, namely: IS*110* (IS*1111* group), IS*1595* (IS*1595* group), IS*200*/IS*605* (IS*200* group), IS*3* (IS*3* and IS*407* groups), IS*481*, IS*5* (IS*5* and IS*903* groups), IS*630*, and Tn*3*. All the transposable elements were distinguished exclusively in the chromosome.

### CRISPR System

Although gene exchange between the bacterial strains is often beneficial, excessive DNA transfer may cause damage to the cells. One of the common defense mechanisms against foreign DNA are elements of the CRISPR system. Within the genome of *Aeromonas* sp. O23A a CRISPR *locus* containing six genes encoding CRISPR-associated proteins and the upstream region containing 54 short spacer sequences (53 of 32 bp in length and 1 of 33 bp) was identified. BLASTN comparative analysis revealed that some of the spacers show sequence similarity to: (i) phages, including *Aeromonas* phage vB_AsaM-56 (Acc no. JQ177063), *Aeromonas* phage DH1 clone SEQDH5 (Acc. no. EU515217) and *Sinorhizobium meliloti* phage PBC5 (Acc. no. AF448724); (ii) plasmids, including *Ralstonia solanacearum* PSI07 megaplasmid mpPSI07 (Acc no. FP885891), *Azospirillum brasilense* Sp7 plasmid ABSP7_p3 (Acc. no. CP012917), *Ensifer adhaerens* OV14 plasmid pOV14b (CP007239.1), *Streptomyces clavuligerus* F613-1 plasmid pSCL4 (Acc. no. CP016560), *Burkholderia rhizoxinica* HKI 454 plasmid pBRH01 (Acc. no. FR687360), *Pseudomonas fluorescens* SBW25 plasmid pQBR103 (Acc. no. AM235768), *Nitrobacter hamburgensis* X14 plasmid 1 (Acc. no. CP000320), *Sphingomonas* sp. NIC1 plasmid unnamed2 (Acc. no. CP015523) and *Rhizobium etli* bv. mimosae strain Mim1 plasmid pRetMIM1e (Acc. no. CP005955); and (iii) genomic islands, including *A. salmonicida* subsp. s*almonicida* JF3224 genomic island AsaGEI2b (Acc. no. KP861348) and *A. salmonicida* HER1085 genomic island AsaGEI1b (Acc. no. KJ626179).

### Heavy Metal Resistance and Metabolism Genes Within the *Aeromonas* sp. O23A Genome

#### Arsenic Metabolism and Resistance

**FIGURE 3:**
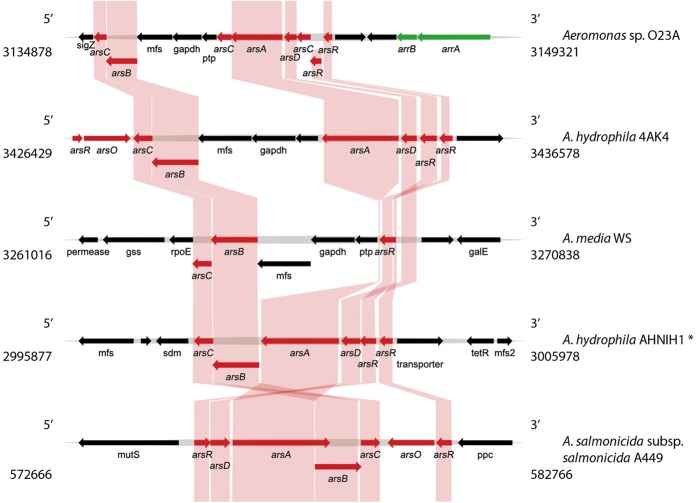
**Comparative analysis of the arsenic resistance and respiration genes (Module 1) found within the O23A chromosome, with the most similar modules found in genomic databases**. Genes encoding proteins involved in arsenic resistance (*ars*) are indicated in red, whereas genes responsible for arsenic respiration ability (*arr*) are green. Homologies are indicated by colored frames. Location of the genome fragments used for the analysis are provided for each analyzed sequence. ^∗^*A. hydrophila* AHNIN1 module was used as a representative of several other *A. hydrophila* strains, whose genomic structure of the *ars* module was highly similar to that of AHNIN1.

**FIGURE 4:**
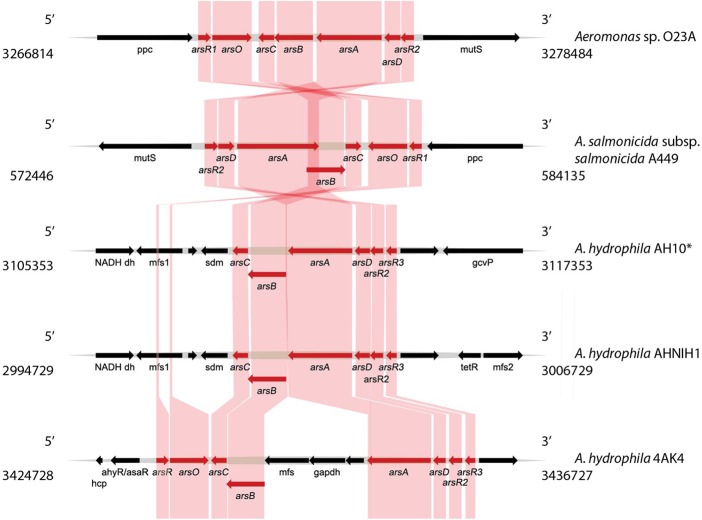
**Comparative analysis of the arsenic resistance genes (Module 2) found within the O23A chromosome, with the most similar modules found in genomic databases**. Genes encoding proteins involved in arsenic resistance (*ars*) are indicated in red. Homologies are indicated by colored frames. Location of the genome fragments used for the analysis are provided for each analyzed sequence. ^∗^*A. hydrophila* AH10 module was used as a representative of other strains *A. hydrophila* AL06-06 and *A. hydrophila* subsp. *hydrophila* ATTC 7966, whose genomic structure of the *ars* module was highly similar to that of AH10.

Bioinformatic analysis of the chromosome sequence of O23A indicated the presence of two genetic modules related to arsenic resistance and metabolism. The first one (Module 1) consisting of a set of *ars* and *arr* genes conferring arsenic resistance and respiration abilities, respectively, is located between 3134878 and 3149321 nt of the chromosome (**Figure 3**). The second arsenic resistance module (Module 2) encoded by the O23A chromosome is located between 3266814 and 3278484 nt (**Figure 4**). It consists of a complete set of *ars* genes, typical for arsenic resistance operon (*arsRDABC*) found in many strains, including *E. coli* (Carlin et al., 1995; Edmundson and Horsfall, 2015), but lacks genes for arsenic respiration. Moreover, it differs from the recognized model arsenic resistance module of *Shewanella* ANA3. On the other hand, the *arr* module of O23A more closely resembles that of ANA3, which comprises *arrA* and *arrB* genes, whose products (ArrA and ArrB proteins, respectively) are both required for respiratory As(V) reduction (Saltikov et al., 2005), especially when arsenate concentrations are low (Malasarn et al., 2004). The potential protein product of the *arrB* gene of O23A is in 60% identical with ArrB protein (AAQ01673.1) of ANA3. The similarity of the putative ArrA protein of O23A to that of ANA3 is not as high. Nevertheless, the functionality of the *arr* module of O23A has been confirmed in the previous studies, where O23A, along with other *Aeromonas* spp. strains isolated from the Zloty Stok mine (Drewniak et al., 2010) was found to dissimilatorily reduce arsenate to arsenite in a respiratory process (Uhrynowski et al., 2017).

The differences in the structure of the Modules 1 and 2 suggest that they are of distinct origin. The *ars* genes in Module 1 do not form a concise operon like in Module 2, but are separated from each other by genes encoding predicted: (i) permease of the major facilitator superfamily (*mfs*), (ii) NAD-dependent glyceraldehyde-3-phosphate dehydrogenase (EC 1.2.1.12, *gapdh*), and (iii) protein-tyrosine phosphatase (*ptp*). Interestingly, Module 1 comprises two copies of the *arsR* gene and three copies of the *arsC* gene, which may indicate recent rearrangements within the O23A chromosome, potentially based on homologous recombination. Moreover, the presence of *mfs*, *gapdh*, and *ptp* genes between the *arsA* and *arsB* genes, also seems to be an insertion. Nevertheless, homology of both the O23A arsenic resistance modules with other *Aeromonas* strains was found.

Comparative analysis of Module 1 with other, most identical gene modules involved in arsenic transformations found in the genomic databases using BLAST is shown in **Figure 3**. Although the analyses indicted similarity of this module to other arsenic modules found in *Aeromonas* spp. genomes, this is only with regard to the *ars* part of the O23A module. No significant similarities covering both the potential *arr* genes have been found, making the O23A module unique among other *Aeromonas* species. The arsenic resistance part of Module 1 of O23A most closely resembles that of *A. hydrophila* 4AK4, as in both the strains, *arsA* and *arsB* genes are separated by *mfs*, *gapdh*, and potentially *ptp* genes. Both the modules also show limited similarity to the partial *ars* module of *A. media* WS, which also includes the *mfs*, *gapdh*, and *ptp* insertion, but lacks the *arsA* gene in close proximity to the other *ars* genes.

In turn, Module 2 of O23A appears to be most closely related to that of *A. salmonicida* subsp. *salmonicida* A449, even though their orientation in respective genomes is different (**Figure 4**). This again suggests a close relationship of O23A with A449. The modules of both the strains include the *arsO* gene encoding a putative monooxygenase, which is not present in several other homologous modules found in several strains of *A. hydrophila*. An exception is the *ars* module of *A. hydrophila* 4AK4, which comprises the *arsO* gene. Interestingly, the 4AK4 module shows homology with both *ars* modules of O23A.

Though differing in orientation, the *ars* modules of *A. hydrophila* AH10 and AL06-06, as well as *A. hydrophila* subsp. *hydrophila* ATTC 7966, have almost identical structure. However, the location of additional copies of the *arsR* gene varies from that of O23A and A449, as in the former both *arsR* gene copies are localized together, whereas in the latter two strains, these genes constitute the boundaries of the module, and they are oppositely oriented. Therefore, the module of 4AK4 seems to be a combination of both types of resistance modules – that of O23A/A449 and those of other *A. hydrophila* strains.

Interestingly, further analysis of the O23A genome revealed another (partial) copy of the *arrA* gene within the chromosome of O23A. It is localized between 2790088–2790975 nt, and in close proximity to this location (upstream and downstream), several proteins possibly involved in the horizontal gene transfer are found (mobile element proteins: 2780913–2779794 nt, 2795165–2795716 nt). The potential protein product of this *arrA* gene copy shows resemblance to *A. salmonicida* subsp. *pectinolytica* 34mel. Comparative analyses revealed that two potential proteins encoded within the 34mel genome may encode ArrB homologs, but their identity with the model respiratory arsenate reductase is low (27–31%). Therefore, it cannot be unambiguously concluded that the 34mel strain is capable of dissimilatory arsenate reduction without physiological studies. Nevertheless, this suggests the potential capabilities of the clinical strains *Aeromonas* spp. to transform arsenic, which yet has to be revealed by *in silico* screening, followed by thorough *in vitro* studies. This result may also indicate that the dissimilatory arsenate reduction ability may either have been lost during genome reduction in pathogenic strains such as *A. salmonicida*, or acquired by O23A from other DARB found within the Zloty Stok mine, e.g., from *Shewanella* sp. O23S (Drewniak et al., 2015) through horizontal gene transfer. However, the latter hypothesis may only be confirmed when the genomes of other DARB from the mine are sequenced.

In the case of O23A, the functionality of the *ars* modules was confirmed by remarkably high resistance to both inorganic arsenic forms As(V) 325 mM and As(III) 10 mM, as indicated in the previous study (Uhrynowski et al., 2017). This was expected, as the strain was isolated from an environment highly contaminated with arsenic. This is also consistent with the presence of two potentially functional arsenic resistance modules, whose protein products facilitate subsequent reduction (ArsC) and active transport (ArsA/ArsB) of arsenate out of the cell.

#### Resistance to Other Heavy Metals

The *Aeromonas* sp. O23A proteome was mapped against the BacMet database (Pal et al., 2014). This allowed for the identification of 98 genes possibly involved in resistance to inorganic ions. Each gene in this pool was manually verified and after deletion of the false positive hits, 15 heavy metal resistance (*hmr*) genetic modules were distinguished, including: (i–ii) O23A_p1143 and O23A_p4209 genes encoding magnesium/cobalt efflux protein CorA, (iii) O23A_p0410 encoding copper homeostasis protein CutC, (iv–vi) O23A_p1375, O23A_p3014 and O23A_p3780 encoding copper-translocating P-type ATPase (which may be involved in copper, lead, cadmium, zinc and mercury transport), (vii–viii) O23A_p2248-2249 and O23A_p2630-2631 encoding cobalt, zinc and cadmium resistance systems (CzcAB), (ix) O23A_p2639-2640 encoding copper resistance system CopAB, (x) O23A_p2978 encoding magnesium and cobalt efflux protein CorB, (xi) O23A_p3017 encoding cobalt, zinc and cadmium resistance protein CzcD, (xii–xiii) O23A_p3309 and O23A_p3510 encoding divalent metal cations (Fe/Co/Zn/Cd) transporters of FieF family.

The functionality of the *hmr* modules found within the O23A genome was assessed by MIC analyses. It was found that apart from high concentrations of As(III) and As(V), the strain shows resistance to at least 1 mM of Cd(II) and Co(II), and – as it was previously observed – to 1 mM of and Zn(II), 2 mM of Cu(II) and 5 mM of Ni(II) (Uhrynowski et al., 2017). This indicates that at least one copy of the aforementioned resistance genes encodes a functional product. However, the strain was found to be incapable of growth in the presence of 1 mM of Cr(VI) or 0.1 mM of Hg(II). This may suggest that the copper-translocating P-type ATPases are not functional, or their affinity toward mercury is limited. This is in contrast to other *Aeromonas* spp., e.g., *A. hydrophila* KT20, which is not only resistant, but is directly involved in Hg(II) reduction (Watts and Lloyd, 2013).

### Siderophore Production and Mineral Dissolution

The use of antiSMASH tool (Weber et al., 2015) enabled the identification of two DNA regions within the chromosome of *Aeromonas* sp. O23A, which contain gene clusters possibly responsible for the synthesis of non-ribosomal peptides (putative siderophores). The first gene cluster (coordinates 1,155,068–1,209,363) encoding non-ribosomal peptide synthetase is most probably responsible for the synthesis of an enterobactin-like siderophore, as 20% of its genes shows similarity to enterobactin biosynthetic gene cluster identified in *Enterobacteriaceae*. Another gene cluster (coordinates 3,834,873–3,882,845) found within the O23A chromosome is composed of genes, of which 90% show similarity the pseudomonine biosynthetic gene cluster. Pseudomonine is an isoxazolidone with siderophoric activity, which was isolated from *Pseudomonas fluorescens* AH2 (Anthoni et al., 1995).

The O23A strain was previously found to be capable of arsenic mineral dissolution (Drewniak et al., 2010) and hydroxamate siderophore production (Uhrynowski et al., 2017). However, the actual *in vitro* activity of the produced siderophores has not been tested. Cultivation of the strain on GASN medium, which lacks iron ions, stimulates bacteria to produce siderophores. Mineral dissolution experiments using filtered culture supernatants showed that after 7 days of incubation, concentration of the dissolved iron ions increased by approx. 2.0 mg/l and 3.0 mg/l for the experiments with arsenopyrite and pyrite, respectively, compared to the control (sterile GASN medium). Therefore, if suitable conditions are provided, the strain can contribute to the extraction and controlled release of heavy metals from mine deposits (solid waste) and therefore bioremediation, through the reduction of their environmental load.

On the other hand, siderophore production ability is regarded as typical for pathogens. By facilitating iron uptake, siderophores enable the growth of the strains during infections, when natural defensive mechanisms of the infected host cause iron ions to become less available to microorganisms, e.g., removed from the bloodstream (Parrow et al., 2013). This, in the light of the aforementioned similarities with the fish pathogen *A. salmonicida*, suggests that O23A genome encodes proteins, which may be involved in pathogenesis. Therefore, the application potential of the strain may be limited to closed bioremediation systems, where appropriate safety measures are taken before the remediated soil/water is released back into the environment.

## Conclusion

Aeromonads are known for their ubiquitous nature, which is to a great extent determined by the carried pool of genes, and especially those found within mobile genetic elements. Genomic research is mostly focused on clinically important determinants of these bacteria, such as antibiotic resistance genes, and much less attention is being paid to other adaptive genes, related to, e.g., heavy metal resistance. Such capabilities often remain undiscovered, even though they may affect the entire biology of the microorganisms, as well as the surrounding environment. This is unfortunate, as the determination of the gene pool of microorganisms can provide particularly valuable and interesting information about their abilities, also those which are not demonstrated during growth under optimum and/or laboratory conditions.

In this work, *Aeromonas* sp. O23A was subjected to *in silico* analyses, the results of which were verified based on *in vitro* studies. Genomic analysis enabled screening for traits of potential significance in arsenic metabolism, while physiological tests allowed to experimentally confirm the functionality of the identified genes and phenotypic modules. Genetic modules coding for arsenic resistance and metabolism have been found, and compared with other closely related strains.

The performed comparative analyses suggest that the complete set of genes for arsenic respiration found in O23A is unique. No other similar *arr* gene modules were found in *Aeromonas* spp. strains, whose genomes had been sequenced. However, it cannot be ruled out, that other Aeromonads may be capable of dissimilatory arsenate reduction, especially as genes encoding potential proteins homologous to ArrA and ArrB were distinguished within other *Aeromonas* spp. The functionality of the indicated proteins has yet to be experimentally determined.

The combined genetic-physiological approach seems to be the best way to screen for and confirm the potential of not only Aeromonads, but also other strains. Based on such analysis, O23A was found to be resistant to several heavy metal ions, and capable of producing siderophores, what indicates that the strain carries genes coding for potentially desirable, and undesirable traits, depending on its application. Due to arsenic transformation abilities, O23A has a great application potential in bioremediation, which may be hindered only by the potential pathogenicity of the strain, which will be assessed in further studies.

## Author Contributions

WU participated in genome assembly and its deposition in the GenBank database, performed bioinformatic analyses of heavy metal resistance and metabolism genes, annotated the plasmids, carried out the physiological experiments, analyzed the data and wrote the manuscript. PD and LDz performed a general analysis of the O23A genome, including the investigation of metabolic pathways and mobile genetic elements. MR performed a complete phage analysis and was involved in article preparation. PK, LL, and DA were involved in DNA sequencing, assembly and genome annotation. LDr is the project manager, and was involved in data consultation. All authors read and approved the final manuscript.

## Conflict of Interest Statement

The authors declare that the research was conducted in the absence of any commercial or financial relationships that could be construed as a potential conflict of interest.

## Footnotes

1 http://blast.ncbi.nlm.nih.gov/Blast.cgi

2 http://phast.wishartlab.com/

3 http://www.ncbi.nlm.nih.gov

4 http://www.uniprot.org/

5 www.ebi.ac.uk/clustalw
