# Supplementary material for: Analysis of the Genome and Mobilome of a Dissimilatory Arsenate Reducing Aeromonas sp. O23A Reveals Multiple Mechanisms for Heavy Metal Resistance and Metabolism
Source: Front Microbiol. 2017 May 29;8:936. doi: 10.3389/fmicb.2017.00936 (PMC5446998; doi:10.3389/fmicb.2017.00936)
Supplement: Supplementary file 2 [file Data_Sheet_2.docx]

Supplementary Material

Analysis of the Genome and Mobilome of a Dissimilatory Arsenate Reducing *Aeromonas* sp. O23A Reveals Multiple Mechanisms for Heavy Metal Resistance and Metabolism

Witold Uhrynowski, Przemyslaw Decewicz, Lukasz Dziewit, Monika Radlinska, Pawel S Krawczyk, Leszek Lipinski, Dorota Adamska, Lukasz Drewniak*

*** Correspondence:** Lukasz Drewniak; ldrewniak@biol.uw.edu.pl

# Supplementary Tables

**Table S1.** COG categories of the proteins of *Aeromonas sp.* O23A.

| **Proteins predicted functions (COG categories)** | **Chromosome** | **p23A_P1** | **p23A_P2** | **p23A_P3** | **p23A_P4** |
| --- | --- | --- | --- | --- | --- |
| A:RNA processing and modification | 1 |  |  |  |  |
| B:Chromatin Structure and dynamics | 1 |  |  |  |  |
| C:Energy production and conversion | 230 |  |  |  |  |
| D:Cell cycle control and mitosis | 47 |  |  | 1 |  |
| E:Amino Acid metabolism and transport | 354 |  |  |  |  |
| F:Nucleotide metabolism and transport | 104 |  |  |  |  |
| G:Carbohydrate metabolism and transport | 231 |  |  |  |  |
| H:Coenzyme metabolism | 183 |  |  |  |  |
| I:Lipid metabolism | 142 |  |  |  |  |
| J:Tranlsation | 256 |  |  |  |  |
| K:Transcription | 261 |  |  | 2 | 1 |
| L:Replication and repair | 131 |  | 1 | 6 | 1 |
| M:Cell wall/membrane/envelop biogenesis | 265 |  |  | 1 | 1 |
| N:Cell motility | 144 |  |  | 1 | 2 |
| O:Post-translational modification, protein turnover, chaperone functions | 167 |  |  |  |  |
| P:Inorganic ion transport and metabolism | 217 |  |  |  | 1 |
| Q:Secondary Structure | 68 |  |  |  |  |
| R:General Functional Prediction only | 286 |  |  |  |  |
| S:Function Unknown | 225 |  |  |  |  |
| T:Signal Transduction | 332 |  |  | 1 |  |
| U:Intracellular trafficing and secretion | 89 |  | 1 | 2 | 9 |
| V:Defence mechanism | 94 |  |  | 2 |  |
| W:Extracellular structures | 43 |  |  |  | 2 |
| X:Phage-derived proteins, transposases and other mobilome components | 25 | 1 |  |  |  |
| Y:Nuclear structure |  |  |  |  |  |
| Z:Cytoskeleton | 1 |  |  |  |  |
| In total | 3897 | 1 | 2 | 16 | 17 |

**Table S2.** CDSs of the plasmid pO23A_p1.

| ORF | Coding sequence  position | | Protein size (aa) | Possible function | ORF | Coding sequence  position | | Protein size (aa) | Possible function |
| --- | --- | --- | --- | --- | --- | --- | --- | --- | --- |
|  | start codon | stop codon |  |  |  | start codon | stop codon |  |  |
| 1 (P1p0001) | 39 | 905 | 288 | hypothetical protein | 5 (P1p0005) | 2708 | 2406 | 100 | hypothetical protein |
| 2 (P1p0002) | 902 | 1093 | 63 | hypothetical protein | 6 (P1p0006) | 3131 | 2709 | 140 | hypothetical protein |
| 3 (P1p0003) | 1332 | 1090 | 80 | putative plasmid replication protein RepB | 7 (P1p0007) | 3498 | 3794 | 98 | transcriptional regulator |
| 4 (P1p0004) | 1644 | 1438 | 68 | hypothetical protein |  |  |  |  |  |
|  |  |  |  |  |  |  |  |  |  |

**Table S3.** CDSs of the plasmid pO23A_p2.

| ORF | Coding sequence  position | | Protein size (aa) | Possible function | ORF | Coding sequence  position | | Protein size (aa) | Possible function |
| --- | --- | --- | --- | --- | --- | --- | --- | --- | --- |
|  | start codon | stop codon |  |  |  | start codon | stop codon |  |  |
| 1 (P2p0001) | 1461 | 1 | 486 | mobilization relaxase | 6 (P2p0006) | 4760 | 3780 | 326 | hypothetical protein |
| 2 (P2p0002) | 1628 | 1458 | 56 | hypothetical protein | 7 (P2p0007) | 5241 | 4771 | 156 | membrane protein |
| 3 (P2p0003) | 1969 | 1628 | 113 | plasmid mobilization relaxosome protein MobC | 8 (P2p0008) | 5679 | 5434 | 81 | hypothetical protein |
| 4 (P2p0004) | 2271 | 1966 | 101 | hypothetical protein | 9 (P2p0009) | 6533 | 5772 | 253 | hypothetical protein |
| 5 (P2p0005) | 3783 | 3289 | 164 | hypothetical protein | 10 (P2p0010) | 7384 | 6530 | 284 | DNA methylase |
|  |  |  |  |  |  |  |  |  |  |

**Table S4.** CDSs of the plasmid pO23A_p3.

| ORF | Coding sequence  position | | Protein size (aa) | Possible function | ORF | Coding sequence  position | | Protein size (aa) | Possible function |
| --- | --- | --- | --- | --- | --- | --- | --- | --- | --- |
|  | start codon | stop codon |  |  |  | start codon | stop codon |  |  |
| 1 (P3p0001) | 180 | 584 | 134 | hypothetical protein | 36 (P3p0036) | 30385 | 30071 | 104 | hypothetical protein |
| 2 (P3p0002) | 1043 | 672 | 123 | hypothetical protein | 37 (P3p0037) | 31050 | 30382 | 222 | lytic transglycosylase |
| 3 (P3p0003) | 1313 | 1116 | 65 | hypothetical protein | 38 (P3p0038) | 31525 | 31340 | 61 | hypothetical protein |
| 4 (P3p0004) | 3592 | 1415 | 725 | DNA topoisomerase III | 39 (P3p0039) | 31761 | 32111 | 116 | transcriptional regulator |
| 5 (P3p0005) | 3911 | 3609 | 100 | conjugal transfer protein | 40 (P3p0040) | 32362 | 32120 | 80 | hypothetical protein |
| 6 (P3p0006) | 4081 | 3908 | 57 | hypothetical protein | 41 (P3p0041) | 33297 | 32434 | 287 | hypothetical protein |
| 7 (P3p0007) | 4643 | 4158 | 161 | lytic transglycosylase | 42 (P3p0042) | 33460 | 33305 | 51 | hypothetical protein |
| 8 (P3p0008) | 5728 | 4736 | 330 | hypothetical protein | 43 (P3p0043) | 33597 | 34142 | 181 | hypothetical protein |
| 9 (P3p0009) | 5961 | 5731 | 76 | hypothetical protein | 44 (P3p0044) | 34266 | 34604 | 112 | hypothetical protein |
| 10 (P3p0010) | 6194 | 5958 | 78 | hypothetical protein | 45 (P3p0045) | 35986 | 36402 | 138 | hypothetical protein |
| 11 (P3p0011) | 6546 | 6205 | 113 | hypothetical protein | 46 (P3p0046) | 36411 | 37181 | 256 | hypothetical protein |
| 12 (P3p0012) | 7273 | 6746 | 175 | hypothetical protein | 47 (P3p0047) | 37370 | 37224 | 48 | hypothetical protein |
| 13 (P3p0013) | 7893 | 7312 | 193 | hypothetical protein | 48 (P3p0048) | 38393 | 38668 | 91 | hypothetical protein |
| 14 (P3p0014) | 8211 | 7921 | 96 | hypothetical protein | 49 (P3p0049) | 38665 | 38802 | 45 | hypothetical protein |
| 15 (P3p0015) | 10120 | 8441 | 559 | hypothetical protein | 50 (P3p0050) | 39602 | 38988 | 204 | hypothetical protein |
| 16 (P3p0016) | 10313 | 10741 | 142 | pilus assembly protein PilV | 51 (P3p0051) | 40365 | 39667 | 232 | hypothetical protein |
| 17 (P3p0017) | 11049 | 10804 | 81 | small distal tail fiber protein | 52 (P3p0052) | 40882 | 40367 | 171 | hypothetical protein |
| 18 (P3p0018) | 12229 | 11327 | 300 | hypothetical protein | 53 (P3p0053) | 41205 | 41555 | 116 | molybdopterin-guanine dinucleotide biosynthesis protein MobC |
| 19 (P3p0019) | 12250 | 12411 | 53 | hypothetical protein | 54 (P3p0054) | 41545 | 44505 | 986 | molybdopterin-guanine dinucleotide biosynthesis protein MobB |
| 20 (P3p0020) | 13096 | 12647 | 149 | hypothetical protein | 55 (P3p0055) | 44758 | 44970 | 70 | hypothetical protein |
| 21 (P3p0021) | 14194 | 13118 | 358 | hypothetical protein | 56 (P3p0056) | 44993 | 45232 | 79 | hypothetical protein |
| 22 (P3p0022) | 15779 | 14106 | 557 | hypothetical protein | 57 (P3p0057) | 45734 | 45555 | 59 | hypothetical protein |
| 23 (P3p0023) | 16029 | 15937 | 30 | hypothetical protein | 58 (P3p0058) | 46016 | 46672 | 218 | hypothetical protein |
| 24 (P3p0024) | 17528 | 16371 | 385 | hypothetical protein | 59 (P3p0059) | 47347 | 46907 | 146 | hypothetical protein |
| 25 (P3p0025) | 19012 | 17531 | 493 | hypothetical protein | 60 (P3p0060) | 47592 | 47368 | 74 | hypothetical protein |
| 26 (P3p0026) | 19961 | 19023 | 312 | hypothetical protein | 61 (P3p0061) | 47806 | 47594 | 70 | hypothetical protein |
| 27 (P3p0027) | 21475 | 19976 | 499 | conjugal transfer protein | 62 (P3p0062) | 48429 | 47803 | 208 | chromosome partitioning protein ParA |
| 28 (P3p0028) | 21833 | 21489 | 114 | hypothetical protein | 63 (P3p0063) | 48986 | 48723 | 87 | integron cassette protein |
| 29 (P3p0029) | 22846 | 21830 | 338 | P-type DNA transfer ATPase VirB11 | 64 (P3p0064) | 49613 | 49407 | 68 | hypothetical protein |
| 30 (P3p0030) | 24078 | 22843 | 411 | conjugal transfer protein | 65 (P3p0065) | 50132 | 49824 | 102 | hypothetical protein |
| 31 (P3p0031) | 24857 | 24075 | 260 | VirB9 conjugal transfer protein | 66 (P3p0066) | 50515 | 50153 | 120 | hypothetical protein |
| 32 (P3p0032) | 25639 | 24857 | 260 | conjugal transfer protein | 67 (P3p0067) | 50804 | 50565 | 79 | hypothetical protein |
| 33 (P3p0033) | 26704 | 25769 | 311 | conjugal transfer protein | 68 (P3p0068) | 51527 | 50862 | 221 | hypothetical protein |
| 34 (P3p0034) | 27370 | 26717 | 217 | P-type DNA transfer protein VirB5 | 69 (P3p0069) | 51804 | 51586 | 72 | hypothetical protein |
| 35 (P3p0035) | 29762 | 27381 | 793 | conjugal transfer protein |  |  |  |  |  |
|  |  |  |  |  |  |  |  |  |  |

**Table S5.** CDSs of the plasmid pO23A_p4.

| ORF | Coding sequence  position | | Protein size (aa) | Possible function | ORF | Coding sequence  position | | Protein size (aa) | Possible function |
| --- | --- | --- | --- | --- | --- | --- | --- | --- | --- |
|  | start codon | stop codon |  |  |  | start codon | stop codon |  |  |
| 1 (P4p0001) | 76 | 195 | 39 | hypothetical protein | 39 (P4p0039) | 22549 | 21620 | 309 | putative plasmid replication protein repA |
| 2 (P4p0002) | 525 | 286 | 79 | hypothetical protein | 40 (P4p0040) | 23191 | 22949 | 80 | hypothetical protein |
| 3 (P4p0003) | 1184 | 540 | 214 | DNA methylase | 41 (P4p0041) | 23754 | 23281 | 157 | hypothetical protein |
| 4 (P4p0004) | 3624 | 1579 | 681 | hypothetical protein | 42 (P4p0042) | 24857 | 23799 | 352 | hypothetical protein |
| 5 (P4p0005) | 3798 | 3995 | 65 | hypothetical protein | 43 (P4p0043) | 25617 | 24886 | 243 | hypothetical protein |
| 6 (P4p0006) | 5015 | 4128 | 295 | transposaseDNA polymerase III, epsilon subunit | 44 (P4p0044) | 25800 | 26573 | 257 | protein KlaA |
| 7 (P4p0007) | 5431 | 5048 | 127 | hypothetical protein | 45 (P4p0045) | 26579 | 27685 | 368 | putative tellurite resistance protein |
| 8 (P4p0008) | 5918 | 5472 | 148 | DNA repair protein RadC | 46 (P4p0046) | 27691 | 28554 | 287 | hypothetical protein |
| 9 (P4p0009) | 6370 | 6008 | 120 | hypothetical protein | 47 (P4p0047) | 28554 | 28904 | 116 | hypothetical protein |
| 10 (P4p0010) | 7055 | 6900 | 51 | hypothetical protein | 48 (P4p0048) | 30126 | 30758 | 210 | LuxR family transcriptional regulator |
| 11 (P4p0011) | 7477 | 7289 | 62 | hypothetical protein | 49 (P4p0049) | 30862 | 30978 | 38 | hypothetical protein |
| 12 (P4p0012) | 7908 | 7711 | 65 | hypothetical protein | 50 (P4p0050) | 31239 | 31547 | 102 | hypothetical protein |
| 13 (P4p0013) | 8800 | 8171 | 209 | hypothetical protein | 51 (P4p0051) | 31657 | 31953 | 98 | type IV conjugative transfer system pilin TraA |
| 14 (P4p0014) | 9072 | 8899 | 57 | hypothetical protein | 52 (P4p0052) | 31972 | 32277 | 101 | conjugal transfer protein TraL |
| 15 (P4p0015) | 9277 | 9140 | 45, 80 | putative recombinase | 53 (P4p0053) | 32280 | 32846 | 188 | protein TraE |
| 16 (P4p0016) | 9520 | 9711 | 63 | hypothetical protein | 54 (P4p0054) | 32914 | 33606 | 230 | conjugal transfer protein TraK |
| 17 (P4p0017) | 10205 | 9792 | 137 | Antitoxin HicB | 55 (P4p0055) | 33599 | 34951 | 450 | conjugal transfer protein TraB |
| 18 (P4p0018) | 10432 | 10256 | 58 | mRNA interferase HicA | 56 (P4p0056) | 34964 | 35518 | 184 | type IV conjugative transfer system protein TraV |
| 19 (P4p0019) | 11183 | 11470 | 95 | hypothetical protein | 57 (P4p0057) | 35529 | 38111 | 860 | type IV secretion system protein TraC |
| 20 (P4p0020) | 11503 | 11718 | 71 | hypothetical protein | 58 (P4p0058) | 38099 | 38536 | 145 | conjugal transfer protein TrbI |
| 21 (P4p0021) | 12093 | 11971 | 40 | hypothetical protein | 59 (P4p0059) | 38706 | 38551 | 51 | hypothetical protein |
| 22 (P4p0022) | 12463 | 12077 | 128 | hypothetical protein | 60 (P4p0060) | 38770 | 39165 | 131 | conjugal transfer protein TraW |
| 23 (P4p0023) | 12890 | 12561 | 109 | hypothetical protein | 61 (P4p0061) | 39165 | 40154 | 329 | conjugal transfer protein TraU |
| 24 (P4p0024) | 13346 | 13092 | 84 | hypothetical protein | 62 (P4p0062) | 40165 | 40761 | 198 | type-F conjugative transfer system pilin assembly protein TrbC |
| 25 (P4p0025) | 13699 | 13544 | 51 | hypothetical protein | 63 (P4p0063) | 40821 | 42707 | 628 | conjugal transfer protein TraN |
| 26 (P4p0026) | 13764 | 14186 | 140 | UV protection and mutation protein | 64 (P4p0064) | 42704 | 43567 | 287 | conjugal transfer mating pair stabilization protein TraN |
| 27 (P4p0027) | 14183 | 15463 | 426 | protein UmuC | 65 (P4p0065) | 43595 | 44125 | 176 | conjugal transfer protein TrbB |
| 28 (P4p0028) | 15873 | 15562 | 103 | hypothetical protein | 66 (P4p0066) | 44112 | 45476 | 454 | conjugal transfer protein TraH |
| 29 (P4p0029) | 16319 | 16002 | 105 | hypothetical protein | 67 (P4p0067) | 45476 | 48313 | 945 | conjugal transfer protein TraG |
| 30 (P4p0030) | 16795 | 16463 | 110 | hypothetical protein | 68 (P4p0068) | 48310 | 48870 | 186 | conjugal transfer entry exclusion protein TraS |
| 31 (P4p0031) | 17067 | 16792 | 91 | hypothetical protein | 69 (P4p0069) | 48901 | 49632 | 243 | conjugal transfer protein TraT |
| 32 (P4p0032) | 17719 | 17246 | 157 | hypothetical protein | 70 (P4p0070) | 49686 | 51833 | 715 | type IV conjugative transfer system coupling protein TraD |
| 33 (P4p0033) | 17975 | 18733 | 252 | Resolvase | 71 (P4p0071) | 51833 | 56959 | 1708 | conjugative transfer relaxase |
| 34 (P4p0034) | 19383 | 18832 | 183 | hypothetical protein | 72 (P4p0072) | 57378 | 57016 | 120 | conjugal transfer protein TraM |
| 35 (P4p0035) | 19441 | 19770 | 109 | hypothetical protein | 73 (P4p0073) | 58037 | 58510 | 157 | lytic transglycosylase |
| 36 (P4p0036) | 20109 | 20357 | 82 | plasmid replication protein RepB | 74 (P4p0074) | 58916 | 58512 | 134 | antirestriction protein |
| 37 (P4p0037) | 20889 | 20467 | 140 | transcriptional regulator | 75 (P4p0075) | 59322 | 59038 | 94 | hypothetical protein |
| 38 (P4p0038) | 21190 | 21339 | 49 | hypothetical protein | 76 (P4p0076) | 59590 | 59333 | 85 | hypothetical protein |

**Table S6.** Genes located within the ФO23A phage.

| ORF | Coding sequence  position* | | Protein size (aa) | Possible function | ORF | Coding sequence  position | | Protein size (aa) | Possible function |
| --- | --- | --- | --- | --- | --- | --- | --- | --- | --- |
|  | start codon | stop codon |  |  |  | start codon | stop codon |  |  |
| 1 (p0822) | 202 (843259) | 707 (842774) | 161 | hypothetical protein (transcriptional regulator) | 26 (p0797) | 19064 (824417) | 19525 (823956) | 153 | head completion protein (GPL) |
| 2 (p0821) | 1725 (841756) | 679 (842802) | 348 | XerD and XerC integrases | 27 (p0796) | 19522 (823959) | 20043 (823438) | 173 | tail completion protein R (GpR) |
| 3 (p0820) | 2844 (840637) | 1780 (841701) | 354 | lysogenic conversion protein, putAbiC | 28 (p0795) | 20040 (823441) | 20726 (822755) | 228 | virion morphogenesis protein (putative tail completion) |
| 4 (p0819) | 3573 (839908) | 2884 (840597) | 229 | repressor protein (CI, HTH_XRE repressor, Peptidase S24 LexA-like protein) | 29 (p0794) | 20731 (822750) | 21855 (821626) | 374 | tail sheath protein |
| 5 (p0818) | 3693 (839788) | 3881 (839600) | 62 | transcriptional regulator (anti-repressor, HTH_XRE) | 30 (p0793) | 21859 (821622) | 22314 (821167) | 151 | tail tube protein |
| 6 (p0817) | 3903 (839578) | 4172 (839309) | 89 | transcriptional regulator | 31 (p0792) | 22318 (821163) | 22527 (820954) | 69 | DksA-like zinc finger protein |
| 7 (p0816) | 4165 (839316) | 4674 (838807) | 169 | phage regulatory protein | 32 (p0791) | 22544 (820928) | 22879 (820602) | 111 | holin |
| 8 (p0815) | 4685 (838796) | 5143 (838338) | 152 | hypothetical protein | 33 (p0790) | 22866 (820615) | 23327 (820154) | 153 | lysin |
| 9 (p0814) | 5210 (838271) | 5398 (838083) | 62 | hypothetical protein | 34 (p0789) | 23324 (820157) | 23767 (819714) | 147 | hypothetical protein |
| 10 (p0813) | 5401 (838080) | 5664 (837817) | 87 | C protein (tanscriptional regulator HTH_XRE) | 35 (p0788) | 23595 (819736) | 23894 (819587) | 99 | hypothetical protein |
| 11 (p0812) | 5667 (837814) | 5840 (837641) | 57 | hypothetical protein | 36 (p0787) | 23891 (819590) | 24151 (819330) | 86 | hypothetical protein (tail assembly chaperone protein; TAC_10) |
| 12 (p0811) | 5837 (837644) | 6034 (837447) | 65 | hypothetical protein | 37 (p0786) | 24193 (819264) | 24336 (819145) | 47 | hypothetical protein |
| 13 (p0810) | 6031 (837450) | 6240 (837241) | 69 | hypothetical protein | 38 (p0785) | 24343 (819138) | 26115 (817366) | 590 | tail tape measure protein |
| 14 (p0809) | 6237 (837244) | 6494 (836987) | 85 | hypothetical protein | 39 (p0784) | 26112 (817369) | 26435 (817046) | 107 | hypothetical protein |
| 15 (p0808) | 6510 (836971) | 6797 (836684) | 95 | hypothetical protein | 40 (p0783) | 26432 (817049) | 27619 (815862) | 395 | Baseplate J-like protein |
| 16 (p0807) | 6794 (836687) | 9073 (834408) | 759 | replication protein A (GPA) | 41 (p0782) | 27612 (815869) | 28295 (815186) | 227 | phage tail protein (Tail_P2_I) |
| 17 (p0806) | 9075 (834406) | 9587 (833894) | 170 | hypothetical protein | 42 (p0781) | 28292 (815189) | 30211 (813270) | 639 | phage tail-collar fibre protein |
| 18 (p0805) | 9966 (833515) | 10955 (832526) | 329 | hypothetical protein | 43 (p0780) | 30216 (813265) | 30944 (812537) | 242 | hypothetical protein |
| 19 (p0804) | 12774 (830707) | 12142 (831339) | 210 | hypothetical protein | 44 (p0779) | 30956 (812525) | 31502 (811980) | 181 | hypothetical protein |
| 20 (p0803) | 13198 (830283) | 12947 (830534) | 83 | hypothetical protein | 45 (p0778) | 31498 (811983) | 33111 (810370) | 537 | hypothetical protein |
| 21 (p0802) | 14273 (829208) | 13257 (830224) | 338 | zinc-finger protein | 46 (p0777) | 33234 (810247) | 33728 (809753) | 164 | hypothetical protein |
| 22 (p0801) | 16090 (827391) | 14270 (829211) | 606 | phage terminase (transcriptional activator Ogr/Delta-like zinc finger) | 47 (p0776) | 33878 (809603) | 34306 (809175) | 142 | Hypothetical protein |
| 23 (p0800) | 16267 (827214) | 17139 (826342) | 290 | portal protein | 48 (p0775) | 35428 (808053) | 34493 (808988) | 311 | HNH nucleases |
| 24 (p0799) | 17149 (826332) | 18216 (825265) | 355 | terminase large subunit | 49 (p0774) | 36537 (806944) | 35458 (808026) | 360 | DNA-cytosine methyltransferase (NaeI) |
| 25 (p0798) | 18220 (825261) | 18948 (824533) | 242 | phage capsid scaffolding protein ((GPO) serine peptidase) |  |  |  |  |  |
|  |  |  |  |  |  |  |  |  |  |

* values in parentheses refer to localization in O23A chromosome

# Supplementary Figures


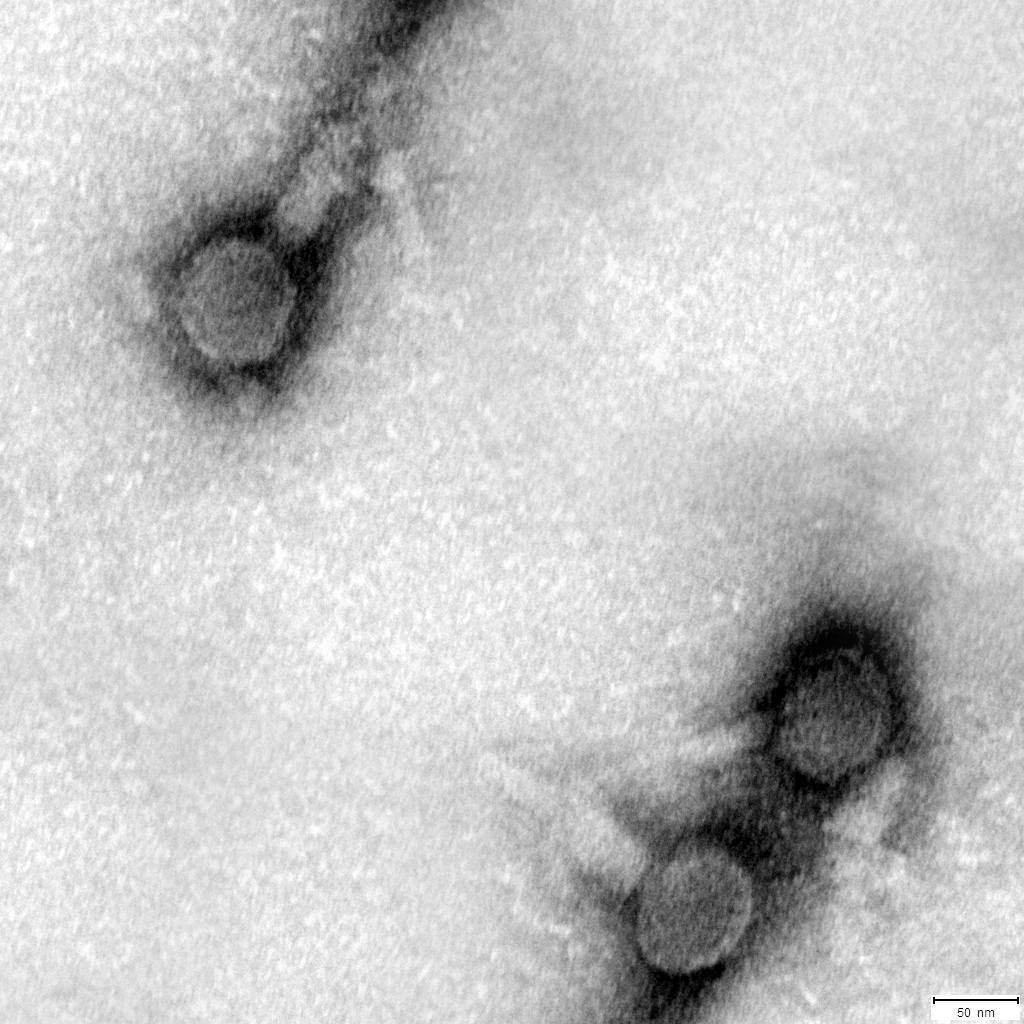


**Figure S1.** Transmission electron microscopy image of the phage ФO23A of *Aeromonas* sp. O23A.
